# Mixed-method analysis of program leader perspectives on the sustainment of multiple child evidence-based practices in a system-driven implementation

**DOI:** 10.1186/s13012-018-0737-6

**Published:** 2018-03-13

**Authors:** Adriana Rodriguez, Anna S. Lau, Blanche Wright, Jennifer Regan, Lauren Brookman-Frazee

**Affiliations:** 1 Department of Psychology, University of California, Los Angeles, California USA; 2 Hathaway-Sycamores Child and Family Services, Pasadena, California USA; 3 Department of Psychiatry, University of California, San Diego, California USA; 4 Child and Adolescent Services Research Center, San Diego, California USA

**Keywords:** Sustainment, Evidence-based practice, Program leader perspectives, children’s mental health services, Mixed-method analysis

## Abstract

**Background:**

Understanding program leader perspectives on the sustainment of evidence-based practice (EBP) in community mental health settings is essential to improving implementation. To date, however, much of the literature has focused on direct service provider perspectives on EBP implementation. The aim of this mixed-method study was to identify factors associated with the sustainment of multiple EBPs within a system-driven implementation effort in children’s mental health services.

**Methods:**

Data were gathered from 186 leaders at 59 agencies within the Los Angeles County Department of Mental Health who were contracted to deliver one of six EBPs within the Prevention and Early Intervention initiative.

**Results:**

Multi-level analyses of quantitative survey data (*N* = 186) revealed a greater probability of leader-reported EBP sustainment in large agencies and when leaders held more positive perceptions toward the EBP. Themes from semi-structured qualitative interviews conducted with a subset of survey participants (*n* = 47) expanded quantitative findings by providing detail on facilitating conditions in larger agencies and aspects of EBP fit that were perceived to lead to greater sustainment, including perceived fit with client needs, implementation requirements, aspects of the organizational workforce, availability of trainings, and overall therapist attitudes about EBPs.

**Conclusions:**

Findings inform EBP implementation efforts regarding decisions around organizational-level supports and promotion of EBP fit.

## Background

Understanding factors associated with the sustainment of evidence-based practices (EBPs) in community mental health settings is essential for leveraging resources in implementation. There have been several large-scale, system-wide EBP implementation efforts focused on improving the overall quality of care in pubic mental health services [1–4]. While these efforts have demonstrated traction in transporting EBPs into settings that can impact public health at the population level, there are limited data on factors that facilitate or hinder the long-term sustainment of EBPs in these settings. Moreover, there are no studies reporting data on long-term viability of scale-up efforts that involve multiple EBPs moved simultaneously into systems of care. A recent systematic review on dissemination and implementation research in children’s mental health reveals that studies on the implementation of single interventions dominate the literature (75% of 80 studies), with few actually focused on long-term sustainment (10%) [5, 6]. Not surprisingly, studies of sustainment have focused on a single innovation within an isolated context [7] or system-wide [8].

Although examining the sustainment of single practices can generate testable hypotheses for improving implementation outcomes, the single practice focus has limited opportunities to study the fit between innovation characteristics and implementation contexts [9] and is inconsistent with the current state of many system reform efforts that involve the dissemination of multiple EBPs to address the major targeted mental health needs of client populations [10–12]. In such system-driven efforts, organizational and leadership factors are likely to be salient drivers of implementation outcomes. Often program leaders (referred to as leaders hereafter) are best positioned to drive change and have profound influence on organizational culture (i.e., workplace norms, provider burnout and priorities) [13, 14]. Yet, a leader’s ability to make change is arguably constrained by the infrastructure of the organization [15, 16]. Identifying organizational and leadership drivers for sustainment of EBPs can inform strategies to improve long-term implementation outcomes, and thus prevent failed efforts to sustain EBPs, reduce financial waste, and maximize public health returns on investments [17].

The process of EBP implementation and sustainment is complex and involves many stakeholders operating at various levels [17–19]. A frequently cited model of implementation processes is the 4-phase EPIS framework (Exploration, Preparation, Implementation, Sustainment) [17], which delineates sustainment as a distinct phase. The EPIS framework addresses both process and context while highlighting the role of inner and outer context factors. The inner context refers to factors within the organization associated with EBP implementation (e.g., program leadership, organizational culture and climate, or characteristics of service providers), while the outer context captures the broader environmental factors that affect operations in a service system such as policies, funding, or system-level leadership [17]. Within system-driven implementation efforts, the outer contextual factors are generally constant across agencies (e.g., contract conditions, reimbursement policies, revenue stream), permitting investigations that can largely isolate determinants of sustainment within the inner context. To date, there has been limited research on inner context leader perspectives on EBP sustainment within system-driven efforts.

Program leaders hold decision-making roles and are tasked with interpreting research evidence and local data, evaluating program performance, and making EBP implementation decisions [20]. Leaders’ attitudes, priorities, and behaviors are major contributors to employee and organizational outcomes [21, 22]. For example, leaders view EBPs more favorably when they perceived their agency as having a high-quality therapist workforce with good capacity for services [23]. Leader perceptions of EBPs are likely crucial in determining the investments made in adopting, supporting, and sustaining EBP delivery. It also stands to reason that leaders with higher roles within the organization (e.g., executive leaders) may hold substantial decision-making power to guide strategy and design of implementation [24, 25]. Yet, no studies have examined the potentially interacting contributions of leader perceptions of EBPs and their decision-making authority as it relates to the sustainment of EBPs. We hypothesized that, within the context of system-driven implementation of multiple EBPs, the probability of sustaining any given EBP is maximized when leaders with the greatest decision-making authority hold favorable views of the innovation.

Studies have also demonstrated the importance of organizational factors on EBP implementation processes [25, 26]. For example, healthy organizational climate (i.e., perceived job autonomy, low stress, expectations for provider knowledge, competence) is associated with positive perceptions of EBPs [26] and reduced turnover [7], all of which can influence sustainment of EBPs. In addition to climate, agency structural characteristics have been linked to sustainment. In a systematic review of factors associated with diffusion and sustainment of innovations in health service settings, organizational size, functional differentiation, and specialization (i.e., number of organizational units and specialties) were associated with innovation adoption but not sustainment [27]. Yet, the authors reasoned that agency size was a proxy for other factors that may relate to capacity for sustainment, such as slack resources [27]. Despite progress in this area, few studies have shed light on associations between organizational characteristics and EBP sustainment within mental health systems.

### Current study

In the present study, we used sequential quantitative and qualitative methods [28] to examine the role of leader perceptions and organizational characteristics that are associated with the sustainment of EBPs within the context of a system-driven implementation in the Los Angeles County Department of Mental Health. The study advances implementation research by examining factors associated with long-term sustainment (i.e., 5 to 6 years following adoption) of multiple EBPs (versus a single innovation). We integrated quantitative and qualitative data for the purpose of triangulation and expansion [28, 29] to provide an in-depth understanding of the associations between EBP sustainment and inner context conditions. First, in our quantitative models of leader survey responses, we predicted that leaders’ positive perceptions of EBPs would be associated with increased probability of practice sustainment, particularly when leaders held executive-level positions. We also examined whether favorable organizational climate (i.e., agency-wide emotional exhaustion, perceived autonomy and involvement) and larger agencies (as a proxy for slack resources) would be associated with EBP sustainment. Using qualitative data from leader interviews, mixed-methods analysis were used to examine convergence with, and expansion of, our quantitative survey findings.

## Method

### Study context

**Fig. 1:**
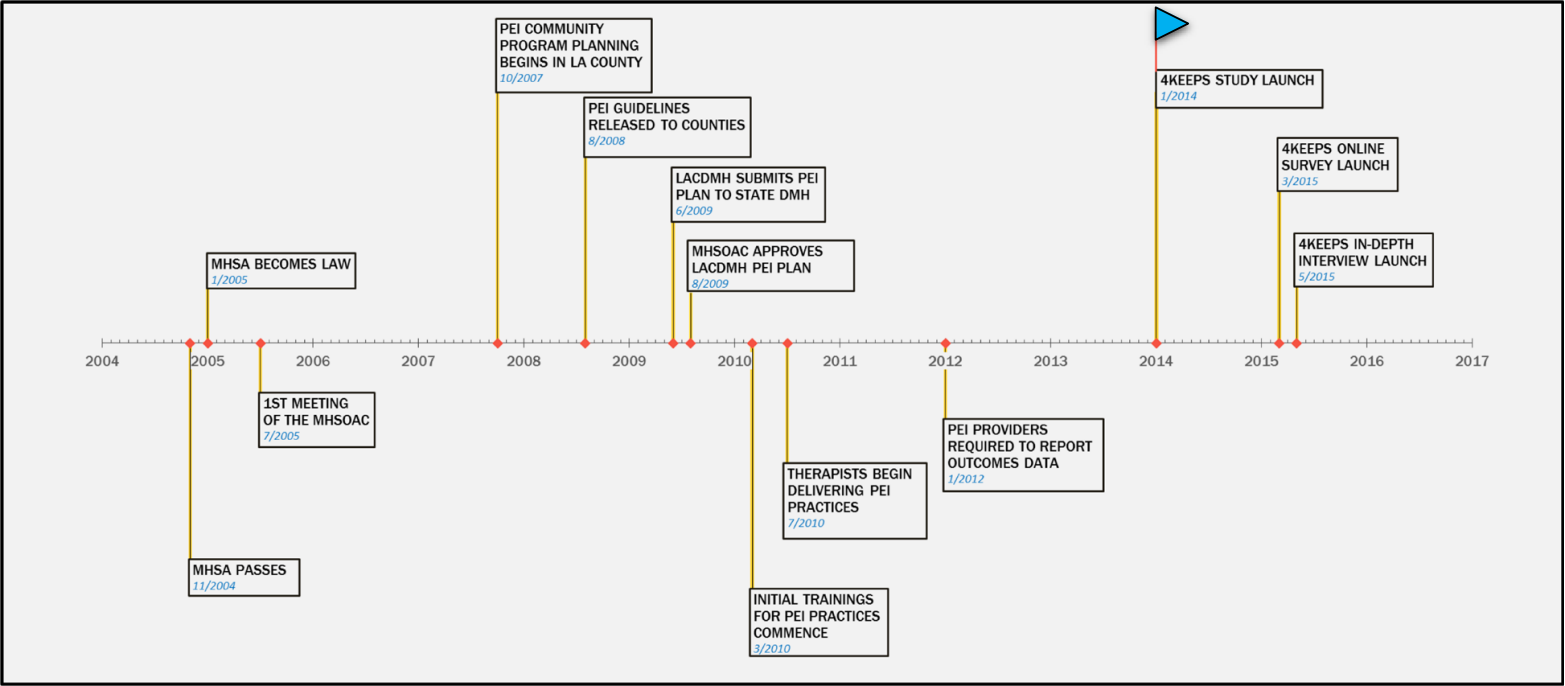
Timeline of major mental health service act- and study-related events. Note: MHSA = Mental Health Services Act; MHSOAC = Mental Health Services Oversight and Accountability Commission; PEI=Prevention and Early Intervention; LACDMH = Los Angeles County Department of Mental Health

This study utilizes both cross-sectional survey and semi-structured interview data from a study examining the sustainment of multiple EBPs within the Los Angeles County Department of Mental Health [10]. The Prevention and Early Intervention (PEI) initiative was funded by a revenue stream from the Mental Health Services Act passed by ballot initiative in 2004. In 2010, PEI promoted the use of EBPs by contracting with community-based agencies to receive reimbursement for approved EBPs. Within children’s mental health services, the County coordinated the rapid scale-up and training of six PEI practices which are the focus of this study [30]: Cognitive Behavioral Interventions for Trauma in Schools (CBITS), Child-Parent Psychotherapy (CPP), Managing and Adapting Practice (MAP), Seeking Safety, Trauma-Focused Cognitive Behavioral Therapy (TF-CBT), and the Triple P Positive Parenting Program (Triple P). A timeline of major events across phases of PEI implementation and study-initiated research activities is presented in Fig. 1. The timing of the data collection allowed us to examine sustainment of the initial six practices several years after adoption.

### Participants and procedures

Ninety-eight agencies that were directly operated or contracted by the Los Angeles County Department of Mental Health to deliver at least one of the six EBPs of interest to children or transition-age youth were eligible for inclusion in the study. We identified eligible participants through agency management at these agencies. Leaders were defined as employees who provided administrative or clinical oversight for at least one of the six EBPs at the agency, while therapists were defined as employees who provided direct service using at least one of the EBPs with youth and families. The research team requested contact information for all eligible staff from the management at the 98 eligible agencies. Contact information for staff from 69 agencies (70.4% of eligible agencies) was obtained for recruitment. Of those 69 agencies, 62 agencies provided email contacts for staff and seven forwarded an email to staff that allowed them to opt-in to provide their email contact to the research team. The county-wide survey was fielded between March 2015 and July 2015 and resulted in 162 leader and 777 therapist participants. The survey response rates for participants recruited from the direct email campaign were 60.7 and 41.5%, for leaders and therapists, respectively. Additional respondents were added during an in-depth study phase that included face-to-face interviews in a subset of 24 programs in 14 agencies [10]. Overall, a total of 186 leader and 824 therapist surveys were included in the present study. Of the full sample of leader survey respondents, 51 leaders also completed the in-depth semi-structured interview focused on their perceptions of the six practices. Respondents received gift cards for completion of the survey and in-person interview ($20 and $40, respectively).

Table 1 shows demographics for all leader participants, including those who participated in the quantitative and/or qualitative components of the study, and therapist participant’s whose survey responses were used in aggregate as indicators of agency-level variables (described under Measures). The average number of participating leaders from each agency was 5.74 (SD = 3.80, range 1–13) for the survey and 4.11 for the interview for leaders (SD = 3.22, range = 1–11). The average number of participating therapists overseen by leaders in each agency was 14.46 (SD = 16.67, range = 1 to 82). Note that there were no statistically significant differences on leader demographic variables between the survey and interview samples.

**Table 1:** Program leader characteristics across survey and interview samples

	Survey (*n* = 186)	Interview (*n* = 51)
	*M* (SD); range or %	
Age	45.09 (9.93); 30–73	44.52 (9.33); 30–78
Female	82.7%	69.2%
Race/ethnicity		
Non-Hispanic White	48.6%	38.5%
Latino/Hispanic	26.5%	30.8%
African-American	7.6%	7.7%
Asian/Pacific Islander	13.5%	19.2%
Multiracial/other	3.8%	3.8%
Licensed	94.6%	94.2%
Degree		
Master’s degree	84.9%	76.9%
Doctoral degree	15.1%	23.1%
Discipline		
MFT	54.6%	53.8%
Psychology	12.4%	17.3%
Social work	33%	28.8%
Leadership level		
Executive	14.6%	15.4%
Middle management	85.4%	84.6%
No. practices adopted^a^	5.70 (2.18); 1–13	5.81 (2.24); 1–10

*Note.* ^a^Average number of practices adopted by agencies from the list of 52 practices approved for reimbursement through the Prevention and Early Intervention initiative

### Measures

#### Qualitative interview

The interview guide focused on perceptions of PEI and the practices being delivered. There were specific questions to gauge barriers and facilitators of implementation of practices (e.g., “What have been challenges associated with implementing [Practice]?”), the impact of adopting (e.g., “How has adopting PEI practices impacted your agency or program?”), and, when relevant, leaders were asked follow-up questions to understand reasons for de-adoption (e.g., “What do you think would have helped your agency implement [Practice]?”).

#### Quantitative survey

All variables were derived from the online survey questionnaire, which asked leaders about their background, agency characteristics, opinions on and experiences with implementing the six practices, and perceptions of support for implementation within their agency.

#### Perceived Characteristics of Intervention Scale (PCIS; [31])

An adapted version of the PCIS was used to examine leader perceptions of the six practices of interest. In relation to each practice, we administered 8 of the 20 original PCIS items related to features of Relative Advantage (e.g., “[Specific practice] is more effective than other therapies used in my program”), Compatibility (e.g., “Using [] fits well with the way therapists in my program like to work.”), Complexity (e.g., “[] is clear and understandable.”), and Potential for Reinvention (e.g., [] can be adapted to meet the needs of our clients.”). Leaders were asked to rate the extent to which they agreed with each item on a 5-point Likert scale (1 = not at all, 5 = to a great extent) for each practice that therapists in their agency had ever used. Leaders reported on an average of 4.22 practices (SD = 1.11, range = 1–6). Consistent with the scoring of the original PCIS, the scale was treated as unidimensional. The mean score of the eight items was used as a total composite score, with higher scores representing more favorable perceptions toward a practice. The total 8-item scale demonstrated strong internal consistency (mean α = .93; range = .91–.97) across practices.

#### Agency and program leader professional characteristics

Questions regarding leader background and professional characteristics (e.g., sociodemographic information, educational training) and characteristics of their agency (e.g., leadership hierarchical) were adapted from previous work [32]. Specifically, we asked leaders to describe to whom they report within their agency and reasoned that the more levels of administrative hierarchy between the leader and the top executive may be an indicator of decision-making authority. We specified leadership level dichotomously as follows: (1) executive leaders: lead executives and those who report directly to the lead executive (i.e., CEO and those one layer below the CEO) and (2) middle management leaders (i.e., leaders with two or more layers below the lead executive).

#### Organizational readiness for change (ORC)

The ORC [33] examines leader perspectives on institutional resources and organizational climate related to the implementation of EBPs. In this study, 10 items from two subscales (i.e., staffing, stress) were used. The staffing subscale measures perceptions of workforce capacity and quality (six items; e.g., “Frequent staff turnover is a problem for your program.”), and the stress subscale measures perceptions of staff strain, stress, and workplace burden (four items; e.g., “The heavy staff workload reduces the effectiveness of your program.”). Leaders rated their agreement with each item on a 5-point Likert scale (1 = disagree strongly, 5 = agree strongly). The mean score of each subscale was calculated based on respective items each with a possible range of 1–5. The subscales demonstrated adequate internal consistency: staffing (α = .63) and stress (α = .83).

#### Therapist autonomy

The Organizational Climate Measure (OCM) examines therapist perceptions of their organization’s policies, practices, and procedures [34]. For the current study, therapists employed at the agency completed the autonomy subscale to describe their perceptions of independence in job-decision making and performance (e.g., “Leaders or agencies keep too tight a reign on the way things are done around here.”). In the current sample, the average number of therapists surveyed was 14.5 (SD = 16.7, range = 1–82). Each item was rated on a 4-point Likert scale ranging from 1 to 4 (1 = definitely false, 4 = definitely true). The mean composite score was calculated with a possible range of 1–4 for each agency. The subscale retained good internal consistency for the current sample (α = .81).

#### Therapist emotional exhaustion

A subset of items from the Organizational Social Context Measure (OSC) [35, 36] was used to assess therapist perceptions of emotional exhaustion in the workplace. Five items from the Emotional Exhaustion subscale of the OSC were used (e.g., “I feel fatigued when I get up in the morning and have to face another day on the job.”). Therapists rated their agreement with each item on a 7-point Likert scale (0 = strongly disagree, 6 = strongly agree). A mean composite score, with a possible range of 0–6, was calculated at the organization-level with higher scores representing more emotional exhaustion among therapist respondents at the agency. The average number of therapists surveyed about emotional exhaustion per agency was 14.5 (SD = 16.7, range = 1–82). In our sample, the measure had strong internal consistency (α = .89).

#### Agency size

Data on agency size were extracted from utilization management reports and was indexed by the average number of child and transition-age youth clients served within the fiscal year from 2011 to 2013 (*M* = 368, SD = 461, range = 0–2347). Agencies were categorized into small (< 100 clients, *n* = 11), moderate (100–500 clients, *n* = 26), and large (> 500 clients, *n* = 15).

### Outcome variable

#### Practice sustainment

Practice sustainment was the outcome of interest for both quantitative and qualitative analysis. We defined sustainment as the continued and current use of an innovation in routine practice [17]. In the survey, leaders reported whether each of the six practices was (a) ever used at the agency (0 = no, 1 = yes) and (b) being used at the agency at the time of data collection (0 = no, 1 = yes). A practice was considered sustained if it was ever used and continued to be used at the time of the survey, and de-adopted if it was used at one time but no longer used presently. We found relatively little variability in reports of practice sustainment between leaders within an agency. A total of 186 leaders reported on six practices, generating 781 practice-specific leader reports on sustainment. Across all reports, over 97% were consistent across leaders within a given agency. The incidents of different program leader reports of practice sustainment within agencies may be explained by different programs retaining certain practices which may not have been sustained agency-wide, and nearly half the agencies in the sample administer multiple programs. Thus, all leader reports were retained for analysis and the sustainment outcome for each practice is measured at the leader level.

Qualitative text was examined to ascertain practice sustainment outcomes based on leaders descriptions of practice-level sustainment. We examined excerpts from interviews wherein leaders described patterns of practice use; decrease in use, de-adoption, increase in use, or stable use of a practice. We classified instances of EBP sustainment (i.e., increased or stable use) versus non-sustainment (decrease in use or de-adoption).

### Data management and analysis

A mixed-method research design was used wherein qualitative semi-structured interview data and quantitative online survey-data were simultaneously collected [28, 37]. For the current analysis, we employed a sequential QUAN ➔ qual mixed-methods to examine factors associated with practice sustainment.

#### Quantitative analyses

A multi-level binomial logistic regression was employed to examine factors associated with a dichotomous practice sustainment variable. The nesting structure consisted of three levels with a total of nine independent variables as follows: (a) level one: leader practice-specific attitudes; (b) level two: the number of practices adopted at the agency, leadership level, the interaction between leadership level and leader practice-specific attitudes, leader perceptions of both the organization’s functioning (e.g., staff turnover) and social context (e.g., staff strain); and (c) level three: agency-level mean of therapist emotional exhaustion and perceived autonomy, and agency size.

#### Qualitative analyses

A “coding, consensus, and comparison” methodology [37] that follows an iterative approach rooted in grounded theory [38] was used to analyze the qualitative interview data. After reviewing a subset of interviews, a scheme of an initial 74 codes was developed and refined by three of the authors (AR, ASL, LBF). Interviews were then independently coded by the coding team of three post-baccalaureate research assistants and a doctoral student.

Finally, to ensure consistency and avoid coder drift, approximately half of the independently coded transcripts were reviewed by the first author and a co-trainer throughout the independent coding process. In instances in which the first author felt that additional or different codes were needed, the reviewer met with the coder and the final codes for that transcript were revised following the consensus discussion. The codes and definitions were refined through this iterative process and resulted in 76 final codes.

#### Integration of data and emergent themes

To examine convergence of findings across both methods, we focused on the extent to which quantitative findings were corroborated by qualitative interviews. We compared emergent themes across instances of EBP sustainment versus non-sustainment. To expand upon the quantitative findings, we explored all possible determinant themes intersecting with sustainment outcome at the practice-leader level.

## Results

### Quantitative results

There were 186 leader participants from 59 agencies with a mean of 3.17 leaders per agency (SD = 2.89, 1–13). There was an average of 2.99 sites per agency (SD = 2.45, 1–9) from the 59 agencies. Table 2 shows descriptive data for practice-specific sustainment across agencies. There was variability in practice sustainment with a range from 46.7% sustainment for CBITS to 100% sustainment for MAP.

**Table 2:** Practice-specific sustainment frequency (%) across agencies (*n* = 59)

Practice	CBITS	CPP	Triple P	SS	TF-CBT	MAP
Ever used *n* (% of sample)	15 (25.4%)	30 (50.8%)	35 (59.3%)	55 (93.2%)	57 (96.6%)	52 (88.1%)
Sustained use *n* (% of ever used)	7 (46.7%)	29 (96.7%)	28 (80.0%)	46 (83.6%)	50 (87.7%)	52 (100%)

The multilevel logistic regression analysis revealed that the expected odds of a leader reporting practice sustainment are decreased by 87% for small agencies in comparison to large agencies (OR = 0.13; 95% CI = [0.03, 0.47], *p = 0.03*). In addition, leaders’ more positive attitudes about practices increased the odds of practice sustainment by 526% (OR = 6.26; 95% CI = [4.12, 9.53], *p < 0.001*). Analysis further indicated that the unique effects of the number of practices adopted, leadership level, leader report of staffing and stress, mean therapist Emotional Exhaustion, and mean therapist autonomy were not significantly associated with practice sustainment. There was also no significant interaction of leadership level and leader perceptions of practices linked to sustainment. See Table 3.

**Table 3:** Multilevel logistic model predicting probability of sustainment of multiple practices

Parameter	Adjusted OR	95% CI	*p*
Intercept	78.20	11.12,549.76	< .001
Time^a^	.89	.59,1.35	0.58
Practice			
PCIS	6.26	4.12,9.53	< .001
Program leader			
No. practices adopted	1.08	.90,1.30	0.41
Executive leader^b^	.46	.13,1.63	0.23
Leadership level × PCIS	1.17	.36,3.87	0.79
ORC staffing	1.67	.78,3.58	0.19
ORC stress	1.39	.86,2.24	0.18
Organization			
Emotional exhaustion^c^	1.88	.72,4.93	0.20
OCM autonomy^c^	2.93	.65,13.27	0.16
Agency size^d^			
Small	.13	.03,.47	0.003
Moderate	.83	.33,2.12	0.69

*Note.* “Multiple” in the title indicates continued use of any of the multiple practices that were initially adopted

^a^Survey completion time difference (log weeks) from start date of dissemination

^b^Middle management leader is the referent group

^c^Therapist aggregated mean

^d^Large agency size is the referent group

### Qualitative results

Descriptions of practice use patterns across all code levels were examined as the primary step for qualitative analysis. There were 51 leader participants from 46 unique agencies (1 small, 19 moderate, 26 large). The single small agency was grouped into the moderate category due to low frequency in the qualitative sample.

Qualitative analysis revealed two themes central to the sustainment of practices: (a) perceptions of fit with client needs, PEI implementation requirements, and organization program mission, and (b) perceptions of organizational context and workforce. Table 4 outlines primary themes and representative quotes.

**Table 4:** Mixed-method results demonstrating conversion and expansion of findings with representative quotes

	Themes	Representative quotes
The importance of fit	1. Fit of practice with client clinical needs	*+* Fit with client needs across symptom, family, ethnic-racial diversity. a. “We don’t see it as fitting in our program because it’s so superficial…not really addressing the issues that we have. And a lot of the time Seeking Safety is linked to substance use and we’re not having a lot of clients disclosing substance use. And even if they are, we have a specialized program we refer them out to for substance abuse treatment.” b. “So Seeking Safety doesn’t really fit age-wise. Especially our more intensive field-based programs. They see younger kids there more seldom, so they use it less.” c. “I would imagine that many of them couldn’t read anything that complicated. So it just didn’t work out.” d. “And you also need a place where – like if you have one child or two children it’s one thing; when you’ve got five kids screaming and yelling at each other, how do you really target the one child who’s our client? It just didn’t work out.” e. “They didn’t wanna watch it [video resources] … It’s white. There is not a single African American person on it. There’s no diversity….it might help if you had a more diverse …, culturally meaningful video to show people that they [clients] could relate to.” f. “Traumas with API [Asian/Pacific Islander] is too [much] full self-disclosure to have in a group setting…so that’s why it’s not working out.”
	2. Fit of practice PEI Implementation requirements	+ Client fit with PEI eligibility requirements. a. “there just weren’t enough clients that met the criteria” b. “So now we’re able to look at what [practice] clients fit, what clients don’t, which is great” + Allowable billing and reimbursements. c. “We’re using it less and less, with the PEI piece, a lot of the clients here have been in treatment for a long time and so then they don’t fit the PEI criteria, and so then we can’t bill to PEI TF-CBT…that’s why it’s used less.” d. “from when I started to now, DMH has gotten stricter in terms of length of sessions. So, people [therapists] are not using it as much. Like in the beginning, I think it was overused, and they were using it for over a year…And now they know it’s six months or less, people don’t wanna’ use it.” + Compliance with training and certification protocols. e. “TF-CBT specifically, there’s not a lot of ongoing support for the model…specific supervision or meetings on ongoing trainings around this particular model. I think it was more like you do the training requirements and then that’s it…” f. “The certification within the year…I think we have definitely tried to abide by those principles more closely than when we first started out.”
	3. Practice modality fit	+ Logistics with group modality. a. “Some staff were recently trained in [another parenting practice], but, again, the logistics of getting groups—they’re hard to get started. So, I think that’s probably why CBITS and Incredible Years have not done as well.” + Client and family disinterest in groups. b. “We had some referrals but actually the biggest concern was that it was going to be in a group and the kids didn’t want to do it in the group or the parents didn’t want them to do it in the group and, rather, wanted individual services.” c. “We tried doing Seeking Safety in group, we ran a couple of groups. They were older kids. They would stick around for a little bit, and then they’d just drop out. We de-adopted that model.”
	4. Therapist attitudes	General and wide-spread attitudes. a. “It TF-CBT was probably used more than in the beginning. I think especially when it was first rolled out. And when staff are first trained, they’re probably excited about doing it and they want to do it right away. And then, I think, after time maybe that excitement fizzles away and they use it a little bit less.” b. “And I think our staff loves CPP. So that helps that you don’t have to talk them into that it’s a good model. They feel like it fits very well with their theoretical orientation. It kind of forced us to do it. We picked enough variety of choices [EBPs] that we could make it work for us without it feeling alien or like we’re being made to do things that don’t fit our kids.” c. “Just the openness of the therapists wanting to learn something new, and the support from upper management has been great with implementing MAP.” + Therapist practice-specific attitudes toward practice. d. “I think those that enjoyed it like it because it’s one of the simple ones.” e. “The thing about MAP is that’s been the one that actually has stuck because of the flexibility.” f. “So, I think it was partly the model Triple P. It is very short-term.”
Organizational context-workforce considerations	5. Developing the workforce	+ Workforce infrastructure and staff turnover. a. “…Initially I had case managers who were trained in the model and they felt quite uncomfortable. They didn’t embrace it. Felt that the model didn’t fit the acuity of the clients that they were seeing at the time…” b. “Well, we lost the three people – there were four of us trained, so we lost three of them. Now we have to get trained again. It’s very difficult when you’re a small agency”. [moderate] c. “The director happened to be not only overseeing the intensive programs but she was overseeing training. So she took over my position. And so when I lost her—she went out of state—I lost the person who was kind of championing for all the directors and everybody. So there goes the groups.” [moderate] d. “we do have turnaround because people get licensed and they have other opportunities that obviously pay more than a non-profit so that’s been huge” [large] e. “I’ve noticed that many people are interested in coming to our agency because we do have a lot of EBPs and they know that there’s training that will be done” [large] + Developing champions/leaders. f. “Getting individuals who love the model, know the model well enough that they become kind of these informal point people that other clinicians can go to them and ask questions about it.” g. “We probably utilize it TF-CBT more because we recognize the need to have champions so that we could train people internally. It [training] was external and now we’ve had, now probably five people trained as champions. That helps.” Strategic professional development. h. “Those that we’ve brought in, we’ve made sure that they’re individuals who truly embrace the model. So, I have got some clinicians that just love Triple P and would love additional training in it. So, they become almost our champions for those causes.” i. “Here there’s no development plan per clinician, but there’s all these weird opportunities…I don’t feel there’s a unified plan that’s related with quality management…So I’m afraid in this agency we have already lost Triple P*.”* j. “Well, I think the clinicians that get trained as supervisors are usually clinicians that we are trying to provide additional professional developmental opportunities for and that’s in an effort to retain them” [large]
	6. Available training and ongoing supports	+ Availability of internal and external training and covering initial training through post-certification supports. a. “I don’t know what happened, but for some reason there’s not as many trainings” and “The decrease Triple P is because of lack of trainings.” b. “If it’s [consultation, train the trainer] not there, they [practices] disappear. It’s like Triple P. Nobody is doing Triple P here anymore. Not because they didn’t like it, but there’s no ongoing structure once you finish the training.” c. “With TF-CBT you don’t have internal trainers, so that also creates a barrier of entrance.” d. “We’ll have the staff demonstrate how ‘people searches’ are working with their clients. For instance, a 12-year-old with anxiety. What’s the gender? Specific ethnicity? Then go over the result and to compare with the treatment model we are providing right now to see if there’s anything they already incorporated or they need to incorporate. After I started, I sort of promoted this idea. I would say we, every one or two months, will have some discussion around this.” e. “We probably utilize it more because we recognize the need to have champions so that we could train people internally. So, it was external and now we’ve had, now probably five people trained as champions that helps” [large] f. “So, they become almost our champions for those causes and, ideally, we want to be able to get individuals who love the model, know the model well enough that they become kind of these informal point people that other clinicians can go to them and ask questions about it…Those that we’ve brought in, we’ve made sure that they’re individuals who truly embrace the model.” [moderate] g. “We also have invested a significant amount of resource here in building the capacity to do internal training in TF-CBT. And we’ve done that because A) we have this deep training commitment, and B) because there’s turnover. And so, if we’re going to continue to provide TF-CBT over time effectively, we need to have a trained workforce who has the knowledge and skills to provide TF-CBT. And it’s most cost effective if we can train and support those people internally’) [large]

Note: + indicates convergence of quantitative data and qualitative themes; [large/moderate] denotes large- or moderately-sized agencies

### The importance of fit

#### Fit of practice with client clinical needs

Leaders consistently discussed the importance of practice fit with client needs across clinical symptoms, client age, caregiver-family characteristics, and ethnic-racial diversity. Leaders frequently talked about the lack of fit between the practice and client symptom presentation (1a) and the mismatch between the practice and the client age range served in the agency (1b). The lack of fit with caregiver-family characteristics was also noted as hindering practice sustainment. In particular, caregiver literacy issues (1c) and challenges associated with delivering parent-focused interventions in disadvantaged household settings were noted as reasons for reduced use of a practice (1d). Others discussed client ethnic-racial demographic mismatch with the practice materials (1e) and challenges with cultural acceptability (1f) as reasons for de-adoption.

#### Fit of practice PEI implementation requirements

Multiple leaders indicated that client eligibility requirements (based on child age, presenting problem, previous use of MH services) restricted which children could receive a practice under PEI in ways that sometimes led to de-adoption (2a), although some informants indicated that eligibility considerations were actually helpful in guiding client-practice assignments (2b). Relatedly, allowable billing and reimbursement requirements were linked to limitations with client eligibility (2c). Moreover, many leaders mentioned that capitated treatment length restrictions were a stimulus for de-adoption of practices (2d). Some respondents noted that limited ongoing supervision and consultation supports post-training resulted in decreased use of the practice over time (2e). Many also stressed that strict provider certification requirements around session and treatment guidelines resulted in many therapists using practices less frequently (2f).

#### Practice modality fit

Leaders talked about the logistical challenges of initiating treatment groups for those practices that called for a group modality (3a). Leaders anticipated client disinterest in a group format as the main reason for de-adoption (3b), while others described having attempted the practice, but de-adopted after clients appeared disengaged in group therapy (3c).

### Organizational context-workforce considerations

#### Therapist attitudes

Leaders primarily discussed changes in therapist attitudes over the course of the PEI Transformation, in addition to attitudes about specific practice characteristics. One leader described therapist initial excitement and later “fizzled” enthusiasm about practice trainings (4a). When discussing successful sustainment of practices, leaders referenced the ease of use of the practice (4b) and flexibility or adaptability of the practices (4c). De-adoption of practices often resulted from dissatisfaction with length of treatment restrictions (4d).

#### Developing the workforce

Leaders talked about the influence of a compatible workforce with the practices in beliefs, goals, and internal expertise. Lacking a workforce member that embraces or “champions” the EBP (5a, 5b) or failing to identify formal “internal champions” for training were perceived to hinder sustainment (5c). An organizational focus on nurturing fit with therapist clinical interests and therapist specialization in a practice seemed beneficial to sustainment (5d). Leaders also highlighted the importance of implementing a unified system that integrates therapist goals and interests to ultimately foster sustainment of practices, as the absence of such a system contributed to practice de-adoption (5e).

#### Available trainings and ongoing supports

A noted challenge for sustainment was the scarcity of practice trainings, specifically access to ongoing supports post-certification to help therapists implement the practices effectively (6a). The absence of these formalized supports (6b) and lack of internal trainers also seemed to impede active use of practices (6c). It is important to clarify that only some practices allowed for use of train-the-trainer models and ongoing consultation supports based on developer procedures. Those who reported increases in use of certain practices, described the development and implementation of ongoing support strategies for successful sustainment of practices. One leader noted that scheduling didactic time for navigating online resources specific to one practice was beneficial for sustainment (6d).

### Integration of quantitative-qualitative findings

We applied two methods to examine convergence and expansion of findings: (a) exploring coded text specific to the main quantitative findings—therapist attitudes toward practices and agency size (“therapist attitudes,” “workforce”) and (b) merging data sets to explore content by agency size (moderate and large). Table 4 illustrates findings across mixed-methods with representative quotes.

Consistent with agency size quantitative findings, leaders from both moderate and large agencies mentioned the importance of having internal practice champions in support of sustainment. Those from large agencies specifically emphasized implementing internal training and child-practice assignment triage systems to facilitate the continued use of practices (6e), while moderate agencies relied on identifying a single, often informal, PEI-specific champion (6f).

The extent to which staff turnover impacted practice sustainment was evident (external employment or internal promotion), but distinct, for both smaller and larger agencies. Leaders discussed turnover challenges associated with the moderate-sized agencies (5b), highlighting the major impacts on sustainment with even small changes to staffing infrastructure (5c). Staff turnover was also a challenge for large agencies (5d), however, agency resources buffered negative impacts on sustainment by promoting the array of training opportunities to new hires (5e) such as training multiple staff on the same EBP (6 g), or by providing quality supervision to retain staff (5j).

Quantitative findings about therapist attitudes converged with leader interviews such that respondents discussed the importance of organization-wide positive perceptions toward the practice (4b) and upper management support and enthusiasm in helping with practice sustainment at the agency (4c).

## Discussion

Using a mixed-method approach, we examined program leader perspectives and organizational-level determinants of the sustainment of multiple EBPs simultaneously implemented in a children’s mental health services system. Quantitative analysis indicated that agency size and leader practice perceptions were related to practice sustainment. The integration of qualitative data allowed us to triangulate and expand on the leader survey findings. We found that there were likely several aspects of leader practice perceptions that were operating to determine sustainment outcomes. Several themes emerged around practice fit with various factors as well as organizational context and workforce characteristics. In addition, qualitative interview data generated hypotheses about what might underlie the association between agency size and practice sustainment. A discussion on the integration and triangulation of findings is presented first followed by the expansion of findings through the emergent qualitative themes.

Themes from qualitative interviews suggest that continued use of practices was more common in large agencies compared to small- and moderate-sized agencies. Continued delivery of a given practice was partially determined by larger agencies being better equipped to implement agency-wide strategies to buffer sustainment challenges in comparison to smaller agencies, which are limited in resources. Although leaders from both moderate and large agencies discussed the importance of having internal trainers and specialized champions, one could speculate that staff turnover is more consequential for sustainment in smaller agencies. Smaller agencies might have fewer staff specialized in practices or lack internal champions that can pick up the slack with turnover. Previous studies have also identified that larger organization size can facilitate innovation [39, 40]. However, one must consider how the present findings fit within the context of a complex community mental health system following the rapid rollout of multiple EBPs. While the mechanism by which agency size is related to practice sustainment is unclear, it is plausible that size is a proxy for more nuanced factors. For example, the interactions between extraneous organizational factors (e.g., available training funds, staff retention rate, or climate) might be drivers for the size-sustainment relationship. We might expect successful sustainment from larger agencies due to the presumed slack in resources [30]. More research is warranted to disentangle the mechanisms underlying this finding.

Mixed-method analyses demonstrated the importance of leader perceptions of practice. For the quantitative findings, more positive leader perceptions of EBPs were related to practice sustainment. This is not surprising given the plethora of studies indicating that positive staff perceptions are pivotal to the implementation and sustainment of EBPs [23, 26]. Overall, this finding underlines the potential role of leaders and their experiences with practices and the long-term sustainment of practices within a program. More research is clearly needed to understand this relationship and to test the impact of leader-focused intervention on climate and long-term sustainment of EBPs.

Qualitative interviews revealed four sub-themes related to sustainment drivers. Factors related to intervention-client fit [17] were the most salient determinants of practice changes in use irrespective of direction (i.e., increase, stable, decrease, de-adoption). Leaders made more references about the decrease and de-adoption relative to the stable or increase use. Consistent with previous research [41], and with what we might expect from leaders given their administrative and/or clinical oversight roles in “ground-level” challenges, they focus on the challenges most proximal to them.

First, and perhaps not surprisingly, qualitative analysis supported the importance of practice fit with sustainment of the practice. The perception of suitability of the practice to the needs of the client, implementation guidelines, and modality with the organization were deemed most important for the sustainment of the practice. This finding is consistent with studies showing that mental health stakeholders at multiple levels value the fit of individual practices with client, therapist, and organization characteristics [30, 42]. Previous empirical and theoretical work also highlights the importance of fit between the intervention model and characteristics of the setting (e.g., client, organization) to the implementation and sustainment of the practice [18, 43, 44].

A second finding that emerged from the interviews centered on the importance of developing a compatible workforce consistent with the practice beliefs, goals, and internal expertise. In particular, many leaders discussed the importance of fostering internal “champions” for practice. This is consistent with EBP implementation conceptual models (e.g., EPIS [17]) and previous studies showing that sustainment of practices is more successful when placed in an organizational culture that highly values EBP use and facilitates team participation [8, 45, 46].

Relatedly, agency staff discussed how a unified system for integrating, and prioritizing, individualized professional goals and training plans for therapists may prevent the de-adoption of practices. The particular methods and mechanisms by which leaders could implement such a system were unclear in the interviews, however, an interpretation can be offered. It is possible that human resource processes and career development plans that are responsive to therapist professional needs may mitigate staff turnover, which, in turn, can result in therapist retention, continued therapist use of practice, maximal return on EBP training resources, and successful sustainment at the agency level. Reductions in staff turnover can be achieved when management decisions are sensitive to staff needs [47] and the climate for implementation is improved when EBP implementation fits with therapist needs [7]. Future implementation research should assess how the focus on therapist professional development within implementation efforts impact sustainment of EBPs at the provider and organizational levels.

A fourth theme from the interviews highlights the importance of training supports in sustainment. Many respondents discussed the evident pitfalls of limited or absent consultation supports following formal trainings for sustainment. Many discussed that sustainment of practices was challenging without implementing “train-the-trainer” models. This suggests that having access to internal trainers likely facilitates addressing EBP implementation challenges and decreases the agency’s dependence on developer supports so that staff could autonomously sustain the practice. A recent study characterizing provider reactions following the PEI Transformation in Los Angeles County also found that providers viewed the lack of consultation supports following trainings as significantly challenging for practice implementation [48]. From the evidence-based training research, we also know that multicomponent training packages are most effective in adoption and implementation of interventions [49, 50]. Interestingly, however, quantitative findings were divergent in that greater organization autonomy was not significant in improving the odds of sustainment. Overall, because training supports continue to be viewed as important several years following the rapid system changes, and the effectiveness of ongoing supports is empirically supported, identifying implementation strategies focused on fostering training supports is critical.

Together, these findings highlight key determinants that provide some direction on potential mechanisms of implementation interventions aimed to facilitate long-term practice sustainment. In particular, the agency size finding points to a clear need for sustainment interventions that support smaller agencies. Although innovative implementation interventions targeting client fit (e.g., “relevance mapping” [51]) and organization context (e.g., network development, leadership; [52, 53]) have successfully improved outcomes, they have predominately focused on the adoption or early implementation phase of an EBP. The current study is novel in that it provides the platform for developing interventions aimed at long-term sustainment. It delineates a clear next step for developing an intervention “package” that targets leaders’ selection of EBPs to facilitate fit beyond client characteristics while still maximizing client coverage and optimizing workforce capacity to foster long-term sustainment.

Some limitations of the present study should be noted. First, while the quantitative survey was comprehensive, some issues identified in the qualitative data were not captured quantitatively (e.g., workforce professional development) and this limited our ability to triangulate all findings. Second, the interview was structured to capture barriers and facilitators of practice implementation. More targeted interview questions around practice sustainment might have yielded additional information. Third, we were limited to only a few leader and organization-level characteristics in the quantitative analysis. Agency size in particular is a very complicated characteristic that should be examined in a variety of ways. It is possible that the size-sustainment relationship might be affected by the operationalization of size. Relatedly, given our smaller qualitative sample, we were limited to interpreting data from predominately moderate- and large-sized agencies. Replication with a larger sample of small agencies is critical. In addition, because leader reports were obtained some years following initial implementation, their retrospective responses might be biased by current agency decisions about EBPs in their organizations rather than reflecting impressions of the practices at the time of implementation. Finally, our recruitment procedures precluded the ability to report on exact response rates for participating therapists and leaders, and the representativeness of our sample compared to all eligible leaders is unknown.

## Conclusion

These findings inform EBP implementation efforts with decisions around organizational-level supports and promotion of EBP fit. Mixed-methods indicated that agency size and attitudes about practice relate to practice sustainment. The agency size finding, in conjunction with the client fit and organization workforce considerations, suggest the need for sustainment interventions that are aimed at supporting smaller agencies through intentional and systematic implementation planning. Future research should focus on identifying strategies to improve directions on potential mechanisms of implementation interventions to best support long-term practice sustainment. Targeting program leaders’ strategic selection of EBPs is important to facilitate fit across client (maximizing client needs), therapist (training, professional goals, experience), and organizational needs (workforce capacity, optimizing inter-agency networks). These advances hold promise to improve EBP implementation, sustainment, and, ultimately, the quality and outcomes of mental health services.

## Abbreviations

CBITS: Cognitive Behavioral Interventions for Trauma in Schools
CPP: Child-Parent Psychotherapy
EBP: Evidence-based practice
EPIS framework: Exploration, Preparation, Implementation, Sustainment
MAP: Managing and Adapting Practice
OCM: Organizational Climate Measure
ORC: Organizational Readiness for Change
OSC: Organizational Social Context Measure
PCIS: Perceived Characteristics of Intervention Scale
PEI: Prevention and Early Intervention
TF-CBT: Trauma-Focused Cognitive Behavioral Therapy
Triple P: Triple P Positive Parenting Program
